# The Ruminant Farm Systems (RuFaS) model is a platform to support future research and actions for sustainable dairy farming

**DOI:** 10.3168/jdsc.2025-0861

**Published:** 2025-10-30

**Authors:** K.F. Reed, J.M. Tricarico, S. HekmatiAthar, J.S. Waddell, D.A. Andreen, K.R. Briggs, A. Liu, N.D. Tomlinson, J. Adamchick, V.E. Cabrera, H. Hu, Y. Gong, G.M. Graef, M.R. Villalobos-Barquero, J.P. Oliver, D.V. Nydam, N. Ayache, L. McClintock

**Affiliations:** 1 KFR Consulting LLC, Sault Sainte Marie, MI 49783; 2 Dairy Management Inc., Rosemont, IL 60018; 3 Department of Animal and Dairy Sciences, University of Wisconsin–Madison, Madison, WI 53706; 4 Department of Animal Science, Cornell University, Ithaca, NY 14850; 5 Fairlife LLC, Chicago, IL 60607; 6 Department of Public and Ecosystem Health, College of Veterinary Medicine, Cornell University, Ithaca, NY 14850; 7 Department of Animal Science, PRO-DAIRY Dairy Environmental Systems Program, Cornell University, Ithaca, NY 14853; 8 National Milk Producers Federation, Arlington, VA 22201

## Abstract

The Ruminant Farm Systems (RuFaS) model is an open-source, modular, whole-farm simulation platform designed to support interdisciplinary research, innovation, and decision making in sustainable dairy production. This review outlines RuFaS structure, functionality, current applications, and potential for further development. Through integration of biophysical modules for animal, manure, soil and crop, and feed storage systems, RuFaS enables comprehensive evaluation of management strategies, environmental interventions, and productivity outcomes in a whole-farm context. The RuFaS model facilitates hypothesis testing, multi-objective analysis, scenario evaluation, and identification of research gaps by simulating interactions and trade-offs across biological, environmental, and management domains. The model's current applications include its integration into the Farmers Assuring Responsible Management Environmental Stewardship program for GHG accounting and its use in evaluating innovations in nutrition, breeding, and manure management technologies. Its modular architecture supports rapid prototyping, modeling at different scales, and uncertainty analysis, making it adaptable to diverse research questions and stakeholder needs. Finally, it highlights the critical role its open-source foundation has for promoting transparency, reproducibility, and collaborative development across disciplines. Its transparent development process, hosted on GitHub under a GPLv3 license, invites contributions from across disciplines and institutions. Scientists are encouraged to explore RuFaS as a tool for advancing their own research, contributing to model development, and engaging in a shared effort to improve the sustainability of dairy systems.

## Highlights

• Integrates animal, manure, crop, and feed processes in an open-source, modular platform.
• Supports assessment of trade-offs and synergies in sustainability strategies.
• Identifies research gaps by revealing downstream effects and system interactions.
• Accelerates scientific innovation and informs industry and policy decisions.

## Graphical Abstract

**Figure:**
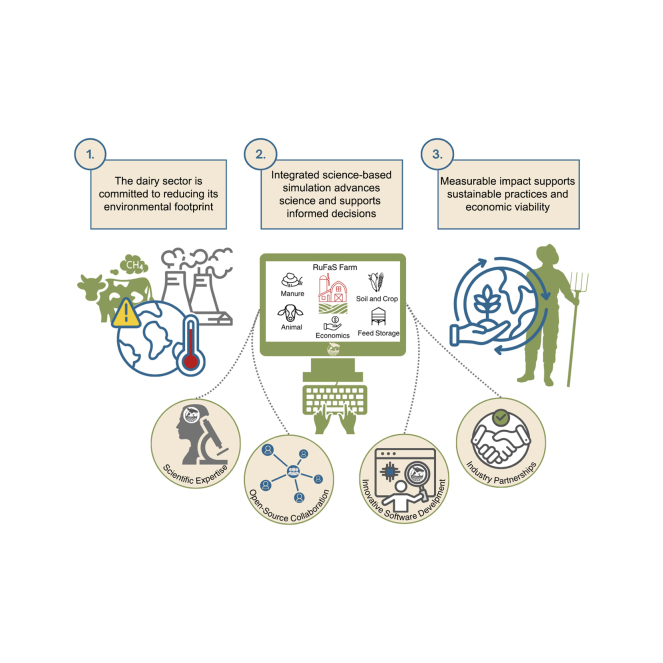
**Summary:** Whole-farm models like the Ruminant Farm Systems (RuFaS) model integrate information to support advances in sustainable dairy research and production. The open-source platform, modern software development, and detailed documentation in the RuFaS modeling environment are designed to foster collaboration and innovation.

Dairy farms are dynamic, complex systems in which changes to one component—such as feed management, herd health, or manure handling—can trigger cascading effects across the entire operation. When research or management decisions are made in isolation, these interdependencies are often overlooked. Whole-farm systems models help reveal these interactions, enabling more informed decisions about resource use, environmental impacts, and economic trade-offs that would otherwise remain invisible in compartmentalized analyses. Agricultural systems models have been in development for decades (Jones et al., 2017a) and there are several existing models capable of representing the whole dairy farm for the purposes of estimating GHG emissions (Rotz, 2018) and other environmental effects. These models produce informative analyses that synthesize current understanding of the physical, management and weather factors driving dairy farm production and environmental impacts (e.g., Veltman et al., 2018; Díaz de Otálora et al., 2024; Godber et al., 2025). The advancement and effects of these models are limited by the state of science and the rate with which they are able to incorporate new scientific knowledge.

Technological advances in computing power, programming languages, and web-based collaboration platforms, create opportunities to build on the intellectual foundation of the existing models while overcoming some of the structural and conceptual limitations highlighted by Antle et al. (2017), Janssen et al. (2017), and Kebreab et al. (2019), such as the lack of integration between parts of the farm, limited or restricted sets of management options, and intractable code bases that restrict the scope and scale of their application and effect. The Ruminant Farm Systems (RuFaS) modeling platform has been in development since 2018 and is designed to meet the growing demand among scientists and dairy sector stakeholders for next-generation, integrative, whole-farm models that facilitate improved and accelerated research to inform coordinated strategies that address sustainability challenges across the dairy supply chain (Table 1).

**Table 1:** Peer-reviewed publications and select proceedings and abstracts reporting development and application of the Ruminant Farm Systems Model

Reference	Publication type	Summary
RuFaS (2025b)	RuFaS documentation	Scientific documentation of the RuFaS Animal Module.
Hu et al. (2025a)	Peer-reviewed manuscript	Illustration of model-based systems engineering application in development of the RuFaS model and case study of the interactions of dairy cow diet, climate, and manure management on greenhouse gas emissions.
Hu et al. (2025b)	Abstract	Preliminary study to evaluate RuFaS barn-floor NH_3_ emission predictions.
Ayache et al. (2025)	Abstract	Summary of methods used to test and integrate RuFaS as the scientific model for FARM-ES.
Gong et al. (2025)	Peer-reviewed manuscript	Development of the method to adjust parity-specific lactation curve parameters in RuFaS to match whole-herd annual milk production.
Weigel et al. (2025)	Peer-reviewed manuscript	Application of the RuFaS model to assess the environmental impacts of genetic selection of dairy cattle.
Reed et al. (2024)	Conference paper	Summary of lessons learned from pilot testing RuFaS on 33 farms for integration with the FARM-ES system.
Li et al. (2023)	Peer-reviewed manuscript	Description and application of the Animal Module to evaluate the effects of reproduction programs on herd level outcomes and profitability.
Gong et al. (2023)	Abstract	Development and preliminary application of RuFaS methods for simulating genetic inheritance.
Li et al. (2022)	Peer-reviewed manuscript	Development of a database of lactation curve parameters for use in the RuFaS model.
Reed (2022)	Conference paper	Application of the RuFaS model to assess the environmental benefits of reproductive efficiency.
Li et al. (2022)	Peer-reviewed manuscript	Development and testing of methods for least-cost diet formulation methods used in RuFaS.
Hansen et al. (2021)	Peer-reviewed manuscript	Initial description and application of the RuFaS Animal Module to quantify the environmental benefits of improved feed efficiency.
Hansen et al. (2020)	Abstract	Development of the methods for maintaining a mass balance of phosphorus within the Animal Module.
Kebreab et al. (2019)	Peer-reviewed manuscript	Review of existing dairy systems models and motivation for a new approach to whole-farm models.

In this mini-review, we describe the significance of systems models for dairy research and industry decision making; summarize the current functionality, applications, and future directions of RuFaS; and highlight the benefits of open-source model development and RuFaS as a platform for open, transdisciplinary, scientific collaboration to advance dairy research and strengthen sustainable dairy production.

Most agricultural research focuses on single system components because rigorous and replicable results necessitate narrowly defined questions. However, the effects of most research findings extend beyond the defined scope of inquiry, and in the context of dairy production, it is difficult to predict and quantify how these effects will percolate throughout the farm without an integrated systems model. The holistic and quantitative outputs of farm-scale models can therefore provide useful feedback at multiple points along the research and development pipeline by helping to identify gaps in knowledge and systems-level trade-offs, providing a virtual platform for hypothesis testing and optimization at different scales and establishing priorities for future work (Figure 1).

**Figure 1:**
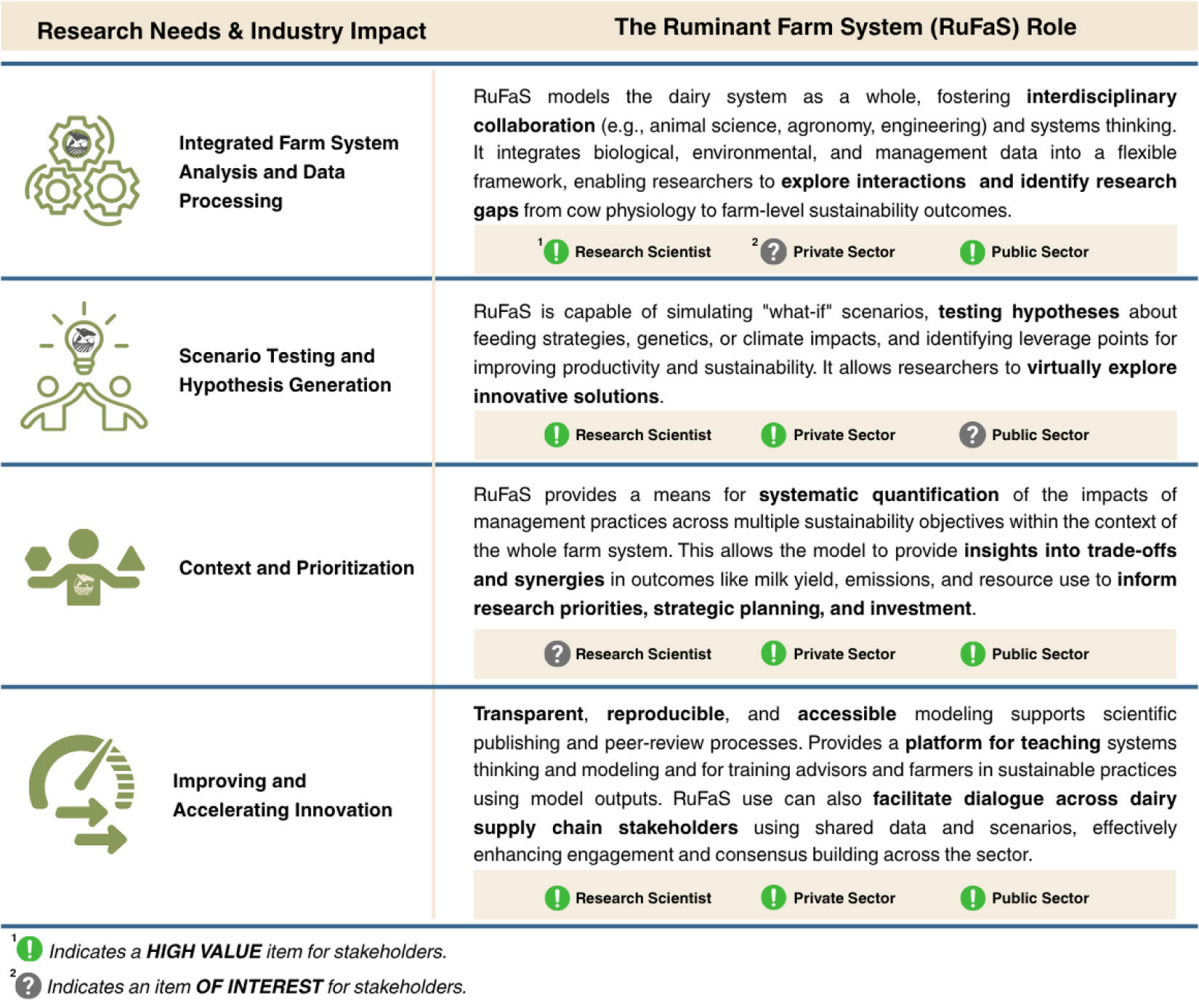
Illustration of research and dairy industry needs for whole-farm systems models, the ways the RuFaS model can meet those needs, and the key stakeholders who will benefit.

Whole-farm, integrated assessments of proposed sustainability interventions are needed to quantify their total and relative effects and identify their trade-offs beyond the scales that can be achieved through empirical research. For example, the evaluation of best management practices by Veltman et al. (2018) using the Integrated Farm Systems Model (**IFSM**; Rotz et al., 2018) could be used to prioritize future research and investment in GHG mitigation programs. This integrated and quantitative assessment identifies feed and manure mitigation strategies as higher priority for research and management intervention to achieve GHG mitigation on dairy farms because they account for more than 90% of the total predicted reduction. Conversely, if the objective is to reduce phosphorus runoff rather than GHG emissions, their analysis points to a focus on field mitigation strategies like no-till and cover cropping. This example illustrates a critical advantage of whole-farm models for multi-objective analysis. By simulating trade-offs and synergies among diverse sustainability goals like GHG mitigation, nutrient management, and productivity, systems models enable researchers and decision makers to evaluate interventions in relation to a broader set of farm and environmental outcomes.

Whole-farm models also provide a low-cost way to scale potential outcomes, develop and test hypotheses, and identify future research needs from preliminary results of novel technologies. For example, feed additives to reduce enteric methane (CH_4_) have been widely studied in lactating cows in confined systems but their effects on young stock, and downstream effects on manure nutrient composition, manure and soil emissions, and crop productivity are largely unknown (del Prado et al., 2025). These knowledge gaps limit the ability to evaluate the effects of interventions for the entire system and constrain efforts to optimize across multiple sustainability objectives, thus emerging as key areas for future research.

Simulating manure excretion and composition across diets and life stages also demonstrates the strategic role of systems models in uncovering research gaps. During RuFaS development, we found wide variation in existing manure excretion prediction methods, especially for calves and heifers, where estimates often rely on BW and intake alone rather than diet composition, thus increasing uncertainty. These limitations, although affecting a small share of emissions, reduce confidence in downstream predictions of water quality and GHG outcomes, emphasizing the need for targeted research. Because integrated analyses that account for downstream effects are key elements of multi-objective sustainability assessments, this underscores the strategic value of integrated systems models not only in identifying research gaps but also in enabling more accurate and precise evaluation of sustainability interventions and in promoting interdisciplinary science.

The advantages of farm systems models for industry and policy mirror the same qualities that make them essential for sustainable dairy research. In particular, the integration of data and processes across the whole farm is a prerequisite for informing decisions related to strategic planning and investment. The ability to conduct benchmarking and comparative analyses of sustainability outcomes at the level of the farm, supply chain, or political region relies on accurate and precise predictions that include both immediate and downstream effects of system changes. Although whole-farm models like RuFaS, IFSM, and COMET-Farm (Paustian et al., 2017; COMET-Farm, 2025) are not designed to directly generate life cycle assessment, inventory, or footprinting results, they play a critical role in supporting those applications by providing detailed, process-based outputs. These outputs must be further synthesized through additional methods, models, or summary analyses to produce the environmental indicators required for most reporting use cases. For example, RuFaS underpins the Farmers Assuring Responsible Management (**FARM**) Environmental Stewardship (**FARM-ES**) program for GHG accounting adopted by processors and cooperatives that make up 80% of the US milk supply. The farm-level outputs from RuFaS provide the majority of inputs for FARM-ES to generate cradle-to-farmgate emissions footprint values for aggregated supply chain reporting by dairy cooperatives, processors, and downstream customers. With the addition of the upcoming Economic Analysis Package, RuFaS integration with FARM-ES will increase the dairy sector's ability to support farmers and cooperatives on their path toward the environmental goals set for 2050 by providing insights into financial sustainability.

Ruminant Farm Systems is a modular, open-source, process-based platform for simulating physical and biological interactions at the farm level. As a whole-farm model, RuFaS predicts the essential outputs required for comprehensive assessment of environmental sustainability, including, milk, meat, and crop production, GHG emissions, ammonia (NH_3_) emissions, water quality impacts, and soil carbon changes, in addition to many more detailed outputs depending on the use case. The core of the simulation model is 4 biophysical (animal, manure, soil and crop, and feed storage) modules that simulate the primary functions of a dairy farm on a daily timestep, including daily exchange of information between modules that reflects the integrated nature of a real dairy farm (Figure 2). Biophysical module execution is coordinated by a simulation engine that receives inputs from a flexible system for input data ingestion and that collects and records extensive, tailored outputs for further processing and analysis. When complete, the Economic Analysis Package will take advantage of these outputs to add economic metrics and comparisons to the sustainability outcomes predicted by the RuFaS model.

**Figure 2:**
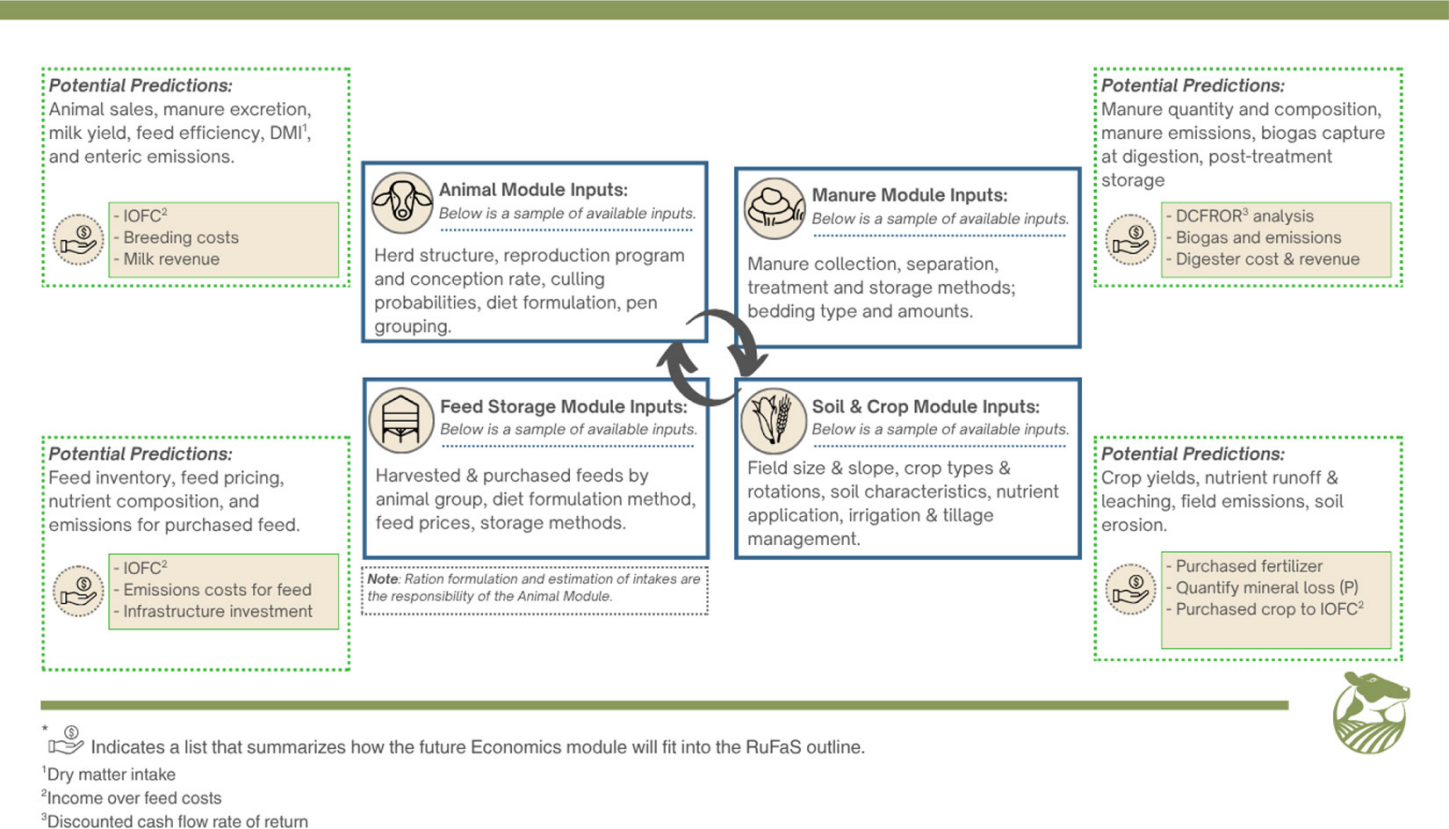
Select inputs, outputs, and processes simulated within the 4 core biophysical modules. A comprehensive description of the inputs and methods can be found in the RuFaS scientific documentation (RuFaS, 2025b).

The “Animal Module” simulates the life cycle and productivity of dairy cattle at both the herd and individual animal levels and is described in detail by Hansen et al. (2021) and Li et al. (2023). It represents 5 life stages, from calf to mature cow, and accounts for reproduction, growth, lactation, and culling events. Herd initialization routines configure herd structure and pen assignments based on user-defined inputs, and herd management functions regulate animal grouping and size over time. Reproductive success and herd exits are determined probabilistically via event-based Monte Carlo simulation driven by user-specified inputs for onset of estrus, conception rate, pregnancy loss, death rate, and so on, as described in Li et al. (2023). Milk production is modeled using dynamic lactation curves (Gong et al., 2025), and diets can be defined by the user or formulated using a least-cost diet optimization algorithm informed by dairy cattle nutrient requirement models (NRC, 2001; NASEM (National Academies of Sciences, Engineering, and Medicine), 2021, Neitsch et al., 2011) to ensure nutritional needs are met (Li et al., 2022). In addition to milk production and feed intake, the module predicts manure and urine output, enteric CH_4_ emissions, and incorporates options for enteric CH_4_ mitigation via 3-nitrooxypropanol or monensin feed additives. Perhaps the most innovative advancement of this module, however, is that it supports flexible herd structures with multiple pens, diverse breeding programs, and culling logic based on fertility and performance.

An example use case of the flexible herd structure is the comparison of different combinations of heifer and cow breeding programs on herd productivity and economic outcomes presented by Li et al. (2023). This work highlights the ability of RuFaS to compare complex and nuanced changes in animal management as illustrated in the 8 scenarios derived through combination of 3 heifer and 4 cow breeding strategies. The benefits of the extensive and diverse outputs produced by the model are also highlighted by the comparison of parity-specific reproduction and culling outcomes and pregnancy survival curves that provide additional context for interpretation of integrated metrics like annual milk production and net return.

Weigel et al. (2025) presented another application of the Animal Module in their assessment of the environmental impacts of genetic selection based on the dairy wellness profit index (**DWP$**). In that work, RuFaS model inputs were developed to represent theoretical herds that matched productivity and health attributes of high and low DWP$ cows from 11 different herds. By replicating the real-world productivity and health outcomes in RuFaS, Weigel et al. (2025) connected and quantified the expected change in environmental outcomes like the enteric CH_4_ emission and N excretion intensity in response to changes in DWP$. This work provides an example of how RuFaS can be used to evaluate the effect of new technologies in cases where we still lack the scientific knowledge to specifically represent that technology in a process-based manner.

Other potential applications of the Animal Module include comparison of different nutritional strategies using the feeds available in the existing feed library, as illustrated by Hu et al. (2024), which compared outcomes from the automated ration formulation under 2 different nutrient requirement models, or by addition of new or novel feeds of interest as long as the required composition inputs can be supplied. There are also opportunities for extension of the first version of the Animal Module to open even further areas for investigation. Some of the future improvements planned for this module include representation of genetic inheritance and updates to the breeding and culling strategies that will expand the available herd management options.

The “Manure Module” represents manure as it moves through collection, storage, and treatment systems. Operating on a daily timestep, it simulates changes in manure mass, nutrient composition, and emissions across customizable manure management chains. The Manure Module includes routines for commonly used storage and treatment systems including solid-liquid separation, slurry storage, anaerobic lagoons, anaerobic digestion (**AD**), bedded packs, open lots, and composting. Each component employs equations specific to its process to estimate outcomes including nutrient losses, CH_4_ production, and NH_3_ volatilization. The module dynamically links to the Animal Module to receive manure excretion data and to the “Soil and Crop Module” for manure application scheduling. The flexible, modular design allows users to aggregate or split manure from different animal housing units in the Animal Module into specified chains that track manure from collection through long-term storage and application to the fields. Each manure management system is itself specified by the user and can be composed of a single or multiple treatment and storage methods sequentially linked.

The Manure Module thus supports holistic assessment of the impact of individual and combined elements of manure management, including trade-offs that might occur between CH_4_ and NH_3_ emission losses at different points along the management chain and nutrient composition at the time of field application. Furthermore, because the module receives manure directly from the Animal Module, it is well suited to assess interactions between diet, manure management, and environment as illustrated by Hu et al. (2025a). This use case compares production and emission outcomes of example farms in 3 different regions under 4 different manure management strategies as an evaluation of the model's ability to meet functional requirements for assessing interactions between diet and manure management. As expected, the results point to the potential of AD for reducing manure CH_4_ emission when compared with slurry storage (0.341 vs. 0.495 kg CO_2_-eq/kg of fat- and protein-corrected milk [**FPCM**]) but also highlight the trade-offs in N loss via NH_3_ volatilization (8.98 vs. 7.38 g/kg of FPCM) in the 2 storage systems, which results from the increase in ammoniacal N concentration in AD digestate. The analysis by Hu et al. (2025a) also contextualizes the relative effects of manure management within the whole-farm footprint with the predicted differences in emissions associated with feed production for the simulated diets exceeding the predicted reductions in manure CH_4_ achieved through AD. Other potential research applications of the Manure Module could compare different manure management scenarios for their effect on whole-farm nutrient efficiency, including variations in timing of manure application, extent of storage emptying, storage type, and use of storage covers. Similar to the Animal Module, the Manure Module has a low barrier for adaptation to test preliminary results from novel technologies like acidification or other manure treatments. Future work planned in this module will update the methods for AD to be responsive to varying management conditions, improve dynamic representation of emissions from solid storage systems, and update the underlying equations for NH_3_ volatilization.

The “Soil and Crop Module” integrates soil biogeochemistry, crop growth, and field management to simulate nutrient dynamics and crop production. Drawing its process representations from 3 existing agronomic and soil biogeochemical models—SWAT (Nietsch et al., 2011), SurPhos (Vadas et al., 2007), and DayCent (Parton et al., 1987)—the Soil and Crop Module simulates soil nutrient cycling, water dynamics, and crop growth under varying management practices, soil conditions, and weather. The module coordinates daily operations such as manure and fertilizer application, tillage, planting, and harvesting across the user-defined number of fields. Soil routines model water balance, erosion, and transformations of nitrogen, phosphorus, and carbon pools, including soil carbon sequestration and GHG emissions. Crop growth is driven by solar radiation and captures biomass accumulation and nutrient uptake under varying conditions related to water, temperature, and nutrient availability. Harvested biomass is transferred to the “Feed Storage Module” for subsequent use in ration formulation, closing the nutrient loop between fields and animals. By representing interactions among soils, crops, and management, this module plays a pivotal role in assessing whole-farm nutrient efficiency and environmental sustainability.

Because the Soil and Crop Module replicates methods from existing models within RuFaS, its primary innovation and opportunity for application is its role in quantifying downstream effects of animal and manure management. Similar to the example of Veltman et al. (2017) discussed earlier, this module is essential for evaluating the relative effects and trade-offs of management strategies across the whole farm. Future work in this module will continue to improve the precision and accuracy of nitrous oxide emissions and soil carbon-sequestration estimates as scientific understanding of these processes and predictive capacity advance. Although current models largely focus on improving the accuracy of crop yield predictions in response to management and environmental conditions, there has been limited progress in linking these drivers to resulting feed composition and nutritional quality (Jones et al., 2017b). Adding this functionality will greatly improve the ability to predict the downstream impact of field management and environment on outcomes like animal feed efficiency and enteric CH_4_ emission.

The Feed Storage Module functions as the central inventory system for harvested and purchased feeds, ensuring that feed availability and quality are accurately tracked throughout the simulation period. This module categorizes feeds into distinct storage classes (grain, hay, silage, and baleage), each subject to characteristic DM and nutrient losses during storage. Losses are calculated using fractional coefficients that reflect storage type and protection level, enabling dynamic adjustments to feed quality over time. Harvested biomass from the Soil and Crop Module and purchased feeds enter the storage system, where nutrient composition is updated to reflect storage effects before being allocated for ration formulation in the Animal Module. By modeling feed losses and inventory management, the Feed Storage Module provides a realistic basis for evaluating ration strategies, feed costs, and their effect on farm productivity and sustainability. There are many opportunities to expand the functionality of this module, including improved process-based representation of spoilage and the effects of inoculants; however, in the context of whole-farm sustainability, the primary benefit of this module is in its ability to complete the nutrient cycle on the farm from crop production to animal feeding and to manage and report daily feed use and inventory.

The proposed “Economics Analysis Package” is under development and will integrate cost and revenue streams associated with milk production, feed procurement, manure handling, and environmental compliance. It aims to simulate profitability under different management scenarios and policy incentives, supporting assessments of economic trade-offs alongside environmental outcomes. When completed, the built-in Economic Analysis Package will allow users to conduct these economic assessments using outputs generated by the RuFaS model directly, rather than requiring an additional post hoc analysis. Although the package will provide sets of default price and cost data, a key feature will be the user's ability to provide their own economic data inputs, so the results represent the current state and specific conditions of inquiry.

The RuFaS model outputs, with the completion of the 4 core process-based biophysical modules described herein as a modular, farm systems modeling framework, now includes estimates of production responses, nutrient cycling, and emissions across the whole farm. These outputs can help scientists explore cross-system interactions of their research, anticipate unintended trade-offs in sustainability metrics, and identify knowledge gaps requiring further empirical study (Figure 1). As early applications illustrate (Table 1), RuFaS transforms the farm into a computational laboratory where scenarios, ranging from genetics and nutrition to manure management and cropping practices, can be digitally and transparently evaluated.

Comparison of RuFaS to existing whole-farm models, such as IFSM (Rotz et al., 2018) and DairyMod (Johnson, et al., 2008) illustrates how RuFaS has drawn from the strengths and improved on shortcomings in other models. First, RuFaS is designed to support modular, multiscale modeling, allowing users to simulate processes at the field, herd, and farm levels independently or in combination. This flexibility is conceptually similar to modularity seen in IFSM and COMET-Farm in that it enables researchers to tailor the model application to specific research questions or data availability, but the combination of object-oriented programming and process-based modeling in RuFaS expands the biological scales that are represented (i.e., from herd to pen or animal) and dynamically shares information between modules to increase understanding of downstream effects. Second, RuFaS enables users to run multiple model configurations or parameter sets in parallel, which enhances the robustness of predictions and supports uncertainty analysis, capabilities that are increasingly important in climate and sustainability research and not available as built-in functionalities of other whole-farm models. Like other whole-farm models, this feature also allows users to define and compare alternative management or policy scenarios within a consistent modeling framework, supporting decision making at multiple levels.

Third, RuFaS's modular architecture and adherence to clean code principles enable rapid prototyping of new modules and functions, unlike legacy systems that are difficult to expand. Furthermore, with modern version-control methods, researchers can develop and test new components like alternative feeding strategies, manure management technologies, or climate scenarios without disrupting the core model. Methods for using and contributing to RuFaS are clearly described on the repository in the README files and supplementary documentation on GitHub pages (RuFaS, 2025a). Fourth, the model includes a built-in data harmonization layer, which standardizes inputs and outputs across modules. This reduces the burden of data preprocessing and lays the groundwork for future interoperability with external datasets and tools for farm data management. Finally, RuFaS includes a transparent and extensible output manager for model diagnostics and visualization that allows users to trace detailed intermediate outcomes that drive model behavior, identify anomalies, and communicate results effectively. These features collectively distinguish RuFaS from other whole-farm models and position it as a flexible, extensible, and scientifically rigorous platform for whole-farm systems modeling.

Commitment to open science and open-source software development drives RuFaS program investment in documentation, selection of the GNU General Public License v3.0 (**GPLv3**), and the mechanisms of distribution. The United Nations Educational, Scientific, and Cultural Organization (**UNESCO**) defines open science as a means “to make multilingual scientific knowledge openly available, accessible, and reusable for everyone, to increase scientific collaborations and sharing of information for the benefits of science and society, and to open the processes of scientific knowledge creation, evaluation, and communication . . . beyond the traditional scientific community” (UNESCO, 2021, p. 7). Similarly, open source is a software development paradigm grounded in the principles of freedom, transparency, and collective advancement. The Open Source Initiative (2006), defines open-source software as freely accessible, modifiable, and redistributable by anyone. The Free Software Foundation (2024) further emphasizes that users should have the freedom to run, study, change, and share software for any purpose.

In the context of agricultural and environmental modeling, combining open science and open-source software development enables researchers to examine and validate model assumptions, adapt tools to local conditions, and contribute improvements that benefit the broader community. It also facilitates integration across disciplines and institutions, increasing collaboration and allowing scientists to build on each other's work. As scientific challenges become more complex and urgent, particularly those related to food systems and sustainability, open-source models offer a pathway to accelerate discovery and democratize innovation.

The RuFaS model adheres to open-source principles through its transparent, community-oriented development process. Under the terms of the GPLv3 license, any modified versions or derivative works must also be distributed under the same license, preserving the freedoms to use, study, modify, and share the RuFaS codebase. In addition, RuFaS includes the GNU Lesser General Public License v3.0 for the use and distribution of its libraries. This allows RuFaS libraries to be dynamically linked with proprietary software without requiring the entire combined work to be open sourced, which is an important consideration for integration into larger systems or commercial applications.

The RuFaS codebase is hosted in a public repository on GitHub (https://github.com/RuminantFarmSystems/RuFaS), where it is actively maintained and openly documented. GitHub is a global communication and version-control platform that enables community-driven development, rigorous code review, and rapid integration of new scientific insights. The mechanisms for coding and noncoding contributions to RuFaS are clearly documented on CONTRIBUTING.md file in the RuFaS GitHub repository root directory (https://github.com/RuminantFarmSystems/RuFaS/blob/dev/CONTRIBUTING.md). This file also includes descriptions of the process for code review and coding conventions, as well as expectations and responsibilities assumed by contributors as their level of involvement increases from RuFaS users, contributors, and sponsors to maintainers and program management leaders. In particular, the RuFaS Program Management Leadership is responsible for providing strategic direction and governance, approving all codebase releases, managing contributor privileges, resolving disputes, and ensuring that RuFaS development aligns with community needs and open-source principles. The RuFaS approach to codebase development, maintenance, and distribution was developed to foster transparency, collaboration, and innovation. Although the repository and its GPLv3 license meet the formal criteria for open-source classification, true accessibility requires that users can understand how to use, modify, and interpret the model. To support this, significant effort was invested in the clarity and availability of the codebase and model documentation. The scientific foundations of the biophysical modules are described in the RuFaS project scientific documentation (RuFaS, 2025b) and are freely available on the repository. This level of transparency allows users to trace model logic, validate outputs, and contribute improvements through version-controlled collaboration.

The RuFaS codebase is written in Python and follows the SOLID (Single responsibility, Open-closed, Liskov substitution, Interface segregation, and Dependency inversion) principles of object-oriented design (see Martin, 2003 and Martin, 2017). This structured approach enhances readability for developers and makes the underlying logic more approachable for subject matter experts without extensive programming experience, while also improving the code's adaptability and ease of maintenance over time. By adhering to this software design philosophy, RuFaS can support active engagement by the scientific community, accelerate model improvement, innovation and discovery and create educational opportunities for students and practitioners.

In summary, RuFaS was designed as a modeling platform for transdisciplinary scientific collaboration. Its open-source foundation and modular design invite researchers from diverse fields to collaboratively create knowledge, test hypotheses, and inform real-world decisions. By integrating biophysical processes with management practices, RuFaS enables holistic assessments of sustainability outcomes across scales and systems. Its accessibility and adaptability make it suitable for a wide range of applications, from academic research and extension to policy analysis and education. Scientists can use RuFaS to explore complex interactions within ruminant farming systems, evaluate the effects of alternative practices, and develop strategies with stakeholders to meet sustainability goals and stewardship commitments across the dairy supply chain. Its transparent development process encourages contributions from across the scientific community, intended to foster a shared sense of ownership and purpose. Through developing new modules, refining existing ones, and applying the model in novel contexts, scientists, educators, and practitioners can contribute to the RuFaS modeling platform as it evolves with the needs of the dairy sector.
